# KRAS Mutation in Serous Borderline Tumor of the Testis: Report of a Case and Review of the Literature

**DOI:** 10.1155/2020/5419707

**Published:** 2020-09-30

**Authors:** Sarah Bouri, Jean-Christophe Noël, Xavier Catteau, Walid Al Hajj Obeid, Ilyas Svistakov, Thierry Roumeguère, Nicky D'Haene, Sandrine Rorive

**Affiliations:** ^1^Department of Pathology, Erasme Hospital, Université Libre de Bruxelles, Brussels, Belgium; ^2^Department of Pathology, Centre Universitaire Inter Regional d'Expertise en Anatomie Pathologique Hospitalière, Jumet, Belgium; ^3^Department of Urology, Erasme Hospital, Université Libre de Bruxelles, Brussels, Belgium

## Abstract

Ovarian-like epithelial tumors of the testis, including serous borderline tumors, are rare entities. We report the case of a 60-year-old man with a left intratesticular mass who had a radical orchidectomy. Histologically, the tumor was identical to the ovarian counterpart showing a well-delineated cystic lesion characterized by intraluminal papillae. The papillae are lined by atypical cuboidal or ciliated cells and are associated with psammoma bodies. The tumor cells express cytokeratin 7 (CK7), cytokeratin 5-6 (CK5-6), cancer antigen 125 (CA125), estrogen (ER), progesterone (PR), Wilm's tumor gene (WT1), paired box gene 8 (PAX8), Ber-EP4, and epithelial membrane antigen (EMA). The diagnosis of a serous borderline tumor of the testis was proposed. Mutation testing using next-generation sequencing showed a Q61K KRAS gene mutation. To the best of our knowledge, this is the second case report of a serous borderline tumor of the testis with a Q61K KRAS gene mutation.

## 1. Introduction

Ovarian Mullerian-like epithelial tumors of the testis are exceedingly rare tumors with about 50 cases described in the literature [1–5]. Like in the ovarian counterpart, serous, mucinous, clear cell, and seromucinous carcinomas have been previously reported [6–8]. The mutational molecular profile of these tumors is poorly understood with only 5 previous cases well documented. In particular, in the serous tumor of the testis, only one previous article had suggested that like in the ovarian neoplasms, KRAS mutation could play a role in the development of these tumors [9]. Here, we report the second case of a serous borderline tumor of the testis in which a mutation of the Q61K KRAS gene is demonstrated. Our data were analysed in the highlight of the literature concerning the molecular profile of this unusual neoplasm.

## 2. Case Presentation

**Figure 1:**
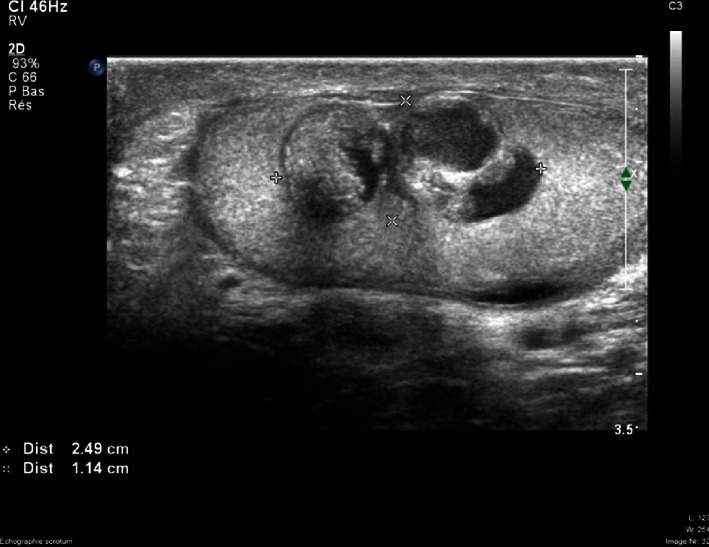
Ultrasound features: heterogenous, hypoechogenic, and cystic tumor of 20 mm.

A 60-year-old man was referred to the urological consultation of Erasme University Hospital for a left intratesticular mass felt by the patient for about six months. At the ultrasound, this tumor of approximately 20 × 13 mm was heterogenous and hypoechogenic and contained calcifications (Figure 1).

**Figure 2:**
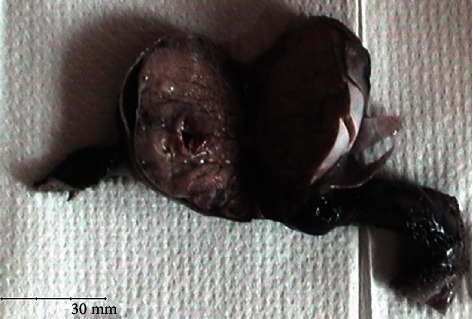
Macroscopically, the intratesticular tumor appeared cystic with an endoluminal solid area.

The epididymis and right testis were normal. Testicular cancer tumor markers such as human chorionic gonadotropin (hCG), alpha-foetoprotein (AFP), and lactate dehydrogenase (LDH) were within normal limits. Therefore, a left radical orchidectomy was performed. Macroscopic examination revealed an intratesticular heterogenous cystic lesion measuring 18 × 13 mm and containing an endoluminal whitish area (Figure 2).

**Figure 3:**
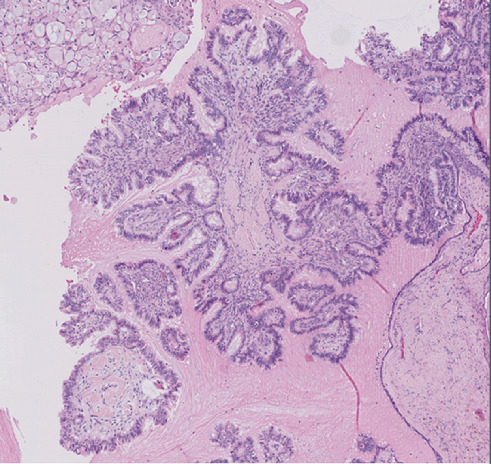
Microscopic view: cystic tumor with papillae lined by pseudo- or pluristratified atypical epithelium (hematoxylin and eosin) (×10).

Microscopically, the tumor was well delineated and characterized by intraluminal papillae. The papillae are lined by pseudo- or pluristratified atypical cuboidal and/or ciliated epithelium. The mitotic index is less than 5 mitoses per HPF (×400). Rare psammoma bodies are also observed. No capsular invasion or vascular involvement is noted (Figure 3).

The tumor cells express cytokeratin-7 (CK7 clone *OV/TL12/30*, 1 : 400, Leica Newcastle, United Kingdom), cytokeratin 5-6 (CK 5-6 clone *D 5/6 B4*, 1 : 100, Dako, Glostrup, Denmark), cancer antigen 125 (CA-125 clone *M1*, ready to use, Dako, Glostrup, Denmark), estrogen (ER clone *EP1*, 1 : 50, Dako, Glostrup, Denmark), progesterone (PR clone *16+SAN27*, 1 : 500, Leica Newcastle, United Kingdom), Wilm's tumor gene (WT1 clone *6F-H2*, 1 : 150, Dako, Glostrup, Denmark), paired box gene 8 (PAX8 clone *MRQ-50*, ready to use, Menarini, Firenze, Italy), Ber-EP4 (clone *Ber-EP4*, 1 : 800, Dako, Glostrup, Denmark), and epithelial membrane antigen (EMA clone E29, 1 : 400, Dako, Glostrup Denmark). We do not observe immunopositivity for cytokeratin 20 (CK20 clone *Ks 20.8*, 1 : 100, Dako, Glostrup, Denmark), CDX2 gene (clone *DAK-CDX2*, 1 : 100, Dako, Glostrup, Denmark), Sal-like protein 4 (SALL4, clone *6E3*, 1 : 100, Cell-Marque, California, United States), human chorionic gonadotropin (beta-HCG clone *GA508*, ready to use, Dako, Glostrup, Denmark), alpha-foetoprotein (AFP clone *GA500*, ready to use, Dako, Glostrup, Denmark), podoplanin (clone D2-40, ready to use, Dako, Glostrup, Denmark), and calretinin (clone *DAK Calret 1*, 1 : 300, Dako, Glostrup, Denmark). p53 protein (clone *DO-7*, 1 : 200, Dako, Glostrup, Denmark) expression is weak and patchy. The proliferative index evaluated by Ki-67 antibody (clone *MIB-1*, 1 : 200, Dako, Glostrup, Denmark) is about 10%.

The molecular profile of the lesion was performed using next-generation sequencing (NGS) with a panel of 16 genes, described in Table 1 [10]. Briefly, DNA was isolated from formalin-fixed and paraffin-embedded (FFPE) tumor samples using the QIAamp FFPE tissue kit (Qiagen, Antwerp, Belgium), according to the manufacturer's instructions. First of all, tumor tissue was manually microdissected with a scalpel on a 10 *μ*m paraffin slide using the H&E-stained slide cut from the same block. This H&E-stained slide was previously reviewed by a pathologist to evaluate the tumor cell percentage in the tumor area. DNA was then quantified using the Qubit® fluorometer and Qubit® ds DNA HS assay kit (Life Technologies, Gent, Belgium). Detection of mutations was performed using a next-generation platform (Ion Torrent, Life Technologies) with a panel of 16 genes (Table 1) previously validated at the Department of Pathology at Erasme Hospital (Brussels, Belgium) [10, 11]. The mutational analysis revealed a Q61K KRAS gene mutation in the testicular tumor.

## 3. Discussion

Ovarian-like epithelial tumors of the testis are extremely rare [1–5]. All types of epithelial tumors, serous, mucinous, seromucinous, and clear cell tumors, have been reported in the testis [6–8]. The most frequently reported types are serous borderline tumors, which share histological and immunohistological similarities with their ovarian counterpart.

These similarities found in such ovarian and testicular serous neoplasms can provide a better comprehension of the pathogenesis of serous borderline testis tumors.

Indeed, several hypotheses are proposed [12]; these tumors may arise from (1) embryological Mullerian remnants in paratesticular tissue, spermatic cord, or epididymis or (2) Mullerian metaplasia of intratesticular inclusions of the tunica vaginalis. This second hypothesis shows a similarity with the origin of the development of the ovarian counterpart, which seems to grow from germinal surface epithelium [13].

However, the mutational profile of such testicular tumors is poorly understood, probably due to the rarity of these entities [9, 12, 14].

We report here a case of a serous borderline tumor of the testis showing a Q61K KRAS gene mutation. This is the second reported case of a KRAS gene mutation in this type of testis tumor. The other gene mutations in these tumors include BRAF gene mutations (Table 2) [9, 12, 14].

Interestingly, in ovarian serous borderline neoplasms, BRAF and KRAS gene mutations are frequently found and known to be involved in the carcinogenesis of these tumors and support common pathogenesis in serous borderline tumors of both female and male, possibly arising from Mullerian origin/remnants [15–17]. BRAF and KRAS are protooncogenes involved in the mitogen-activated protein kinase (MAPK) pathway. The activation of this pathway involves the cascade of activation of signaling proteins such as RAS, RAF, MEK, and ERK, which leads to increased cell growth and differentiation. Mutations in BRAF or KRAS gene result in the constitutional activation of the MAPK pathway and aberrant cell proliferation [18, 19].

Low-grade serous lesions of the testis and their ovarian counterpart have the potentiality of recurrence and metastasis after surgery [20, 21]. Classically, this kind of tumors has shown a low response rate to cytotoxic chemotherapy [20–22]. Therefore, the RAS/RAF/MEK/ERK pathway could constitute an attractive target for cancer drugs in patients with KRAS and/or BRAF mutations [23].

Naturally, due to the rarity of these tumors in testis, their mutational status should be confirmed in the future in a larger series.

## Figures and Tables

**Table 1:** Cancer hotspot panel used by next-generation sequencing.

AKT1	DICER1	FOXL2	POLE
BRAF	ERBB2	KRAS	PTEN
CDKN2A	FBXW7	PIK3CA	RB1
CTNNB1	FGFR2	PIK3R1	TP53

**Table 2:** Summary of gene mutations described in the serous borderline tumor of the testis.

	Gwiti et al., 2017 [9]	Burger et al., 2015 [12]	Cundell et al., 2015 [14]
Mutational analysis (number of cases)	7	2	1
No mutation	4	1	0
KRAS gene mutation	1	0	0
BRAF gene mutation	3	1	1
